# Compensation patterns following occupational injuries in Zambia: results from the 2009 Labour Survey

**DOI:** 10.1186/1755-7682-3-19

**Published:** 2010-09-08

**Authors:** Seter Siziya, Adamson S Muula, Amanda Ryan, Emmanuel Rudatsikira

**Affiliations:** 1 Department of Community Medicine, School of Medicine, University of Zambia, Lusaka, Zambia; 2 Department of Public Health, Division of Community Health, College of Medicine, Blantyre, Malawi; 3 WAHSA programme and Department of Occupational and Environmental Health, School of Public Health, Nelson R Mandela School of Medicine, University of KwaZulu-Natal, SA; 4 Division of Epidemiology and Biostatistics, Graduate School of Public Health, San Diego State University, California, USA

## Abstract

**Background:**

Occupational injuries have received limited research attention in the Southern African Development Community. Much of the published data come from South Africa and little has been reported elsewhere within the region. The present study was conducted to estimate the prevalence rates of occupational injuries and compensation; and to determine factors associated with occupational injuries and compensation.

**Methods:**

Data were obtained from occupational health and injury questions added to the Zambian Labour Force Survey of 2009 by the Work and Health in Southern Africa programme. Logistic regression analyses were conducted to determine the degree of association between demographic, social and economic factors on one hand and injury and compensation on the other.

**Results:**

Data on 61871 study participants were available for analysis, of whom 4998 (8.1%) reported having been injured (10.0% of males, and 6.2% of females) due to work in the previous 12 months to the survey. Of those injured, 60.5% reported having stayed away from work as a result. The commonest type of injury was "open wound" (81.6%). Male gender, being married or married before, being a paid employee, working for a private company and household were positively associated with serious injuries. Injuries also varied by geographical area. Factors positively associated with receiving compensation for work-related injuries were: male gender, Copperbelt and North-Western provinces, and unpaid family worker. Employer/self employed and having less than 5 employees in a workplace were negatively associated with compensation.

**Conclusion:**

The prevalence of reported injury and its association with a significant level of absence from work, indicate that occupational hazards in Zambia have significant health and economic effects. Female workers should equally be compensated for injuries suffered as their male counterparts.

## Background

The Central Statistical Office (CSO) estimates that of all persons employed in Zambia, 72% are engaged in the agriculture sector; 83% are engaged in the informal sector and of all persons engaged in informal sector, 77% are in informal agriculture sector, mainly subsistence farming [1]. Common crops grown include maize, groundnuts, cotton and tobacco. Tobacco and cotton are prone to pests and pesticides are used to control them. Hence most workers in Zambia would suffer from agriculture-related injuries that include: pesticide poisoning [2] and backache or muscle pain [3].

Zambia is a major producer of copper in the world, and the mining sector employs about 15% of formally employed workers [4]. People who work in mines experience various injuries such as noise-induced hearing loss [5,6] caused mainly by blasting and drilling rocks; and head injuries as a result of rock falls and blasts.

The law is in place in Zambia for workers injured at workplaces to claim compensation. Zambia ratified in 1964 the following International Labour Organization (ILO) Social Security Conventions: Convention No. 12 (Workmen's Compensation in agriculture, 1921 [Revised by convention No. 121]; Convention No. 17 (Workmen's Compensation for accidents, 1925 [Revised by convention No. 121]; Convention No. 19 (Equality of treatment for national and foreign workers as regards workmen's compensation for accidents, 1925); and in 1965, Zambia ratified Convention No. 18 (Workmen's compensation for occupational diseases, 1925). The Employment Injury Scheme in Zambia is administered by the Workmen's Compensation Fund Control Board; and the Ministry of Labour and Social Services generally supervises the fund [7].

Research on occupational injuries and the pursuit of improved occupational health has largely been conducted in high- and middle-income nations. Routinely collected occupational health data using information systems from low-income nations are often unavailable or incomplete and unreliable. Much of the data on occupational health and safety from the Southern African Development Community (SADC) which comprise of Angola, Botswana, Democratic Republic of Congo, Lesotho, Madagascar, Malawi, Mauritius, Mozambique, Namibia, Seychelles, South Africa, Swaziland, Tanzania, Zambia and Zimbabwe are from South Africa. There is a paucity of data from the rest of the region. Hence the negative impact of poor work conditions is unappreciated and the scientific basis for interventions and policy formulation is to a great extent absent in this region.

What data there is in Zambia often arises from research done on selected work force populations: in a most recent paper on occupational injuries limited to persons working in mines in Zambia, Michelo et al [4] reported that the most frequent mechanism of injury was handling of tools and materials, and concluded that the fatality rate was higher than that reported in developed countries. However in a nationally representative survey, CSO and the Ministry of Labour and Social Security (MLSS) [8] reported in 2005 that 20.6% of currently employed persons of age 15 years or older reported work-related injury, with 61.6% having suffered back/muscle pain, 20.3% wounds/deep cuts. 14.2% sight problems, and 3.9% hearing problems. These data suggest that workers in Zambia face poor working conditions.

Information on compensation as a result of work-related injury was not covered in the 2005 Labour Force Survey (LFS). Furthermore, no analysis to characterize persons who reported injury was done. Identification of factors associated with injury is important in the design of interventions to control injuries. Hence, using data from the most recent 2009 LFS, we carried out this study to determine the prevalence rates of occupational injuries and compensation; and to establish factors associated with occupational injuries and compensation.

## Methods

### Study design and setting

The Zambia LFS is conducted, as a nationally representative survey using a series of cross sectional studies over time. It was designed to measure the labour market and to provide key indicators of the labour market such as unemployment, underemployment, and hours of work.

The Work and Health in Southern Africa (WAHSA) programme was a 4-year, Swedish-Southern African OHS programme. Among its objectives was the profiling of occupational health and safety (OHS) in the region and, for this purpose, the generation of data to supplement the scarce data available. By an agreement between WAHSA and CSO, a set of OHS questions was added to the 2009 LFS.

### Sample size and sampling

**Figure 1:**
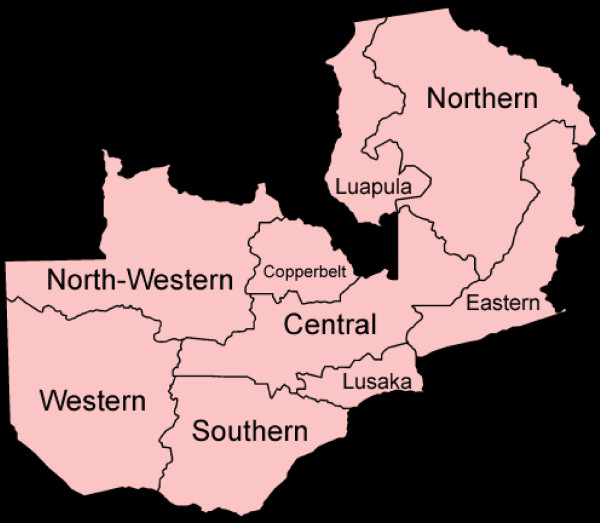
**Map of the Republic of Zambia showing provinces**.

The LFS aims to enroll 8000 households countrywide and the sample size is determined in such a way as to have adequate power to produce estimates for the entire country, urban and rural areas, and for each province. Zambia has nine provinces which are: Central, Copperbelt, Eastern, Luapula, Lusaka, Northern, North-Western, Southern and Western (See Figure 1). The Copperbelt, as the name implies, is the seat of Zambia's copper mining efforts. Employees in Lusaka, the capital city, are mainly engaged in administrative work. Employees in other provinces are mainly engaged in agriculture.

A two stage cluster sampling technique is used to draw sampling units. The primary sampling units are Standard Enumeration Areas (SEAs), identified from a sampling frame compiled from the 2000 population and housing census. In the second stage of sampling, households are systematically sampled in each selected SEA and stratified by urban and rural areas.

### Questionnaire

The composition of the LFS questionnaire varies from survey to survey. In 2009, CSO incorporated OHS questions in the questionnaire on: health outcomes, work sector and conditions, work place facilities, work-related injuries and history of compensation as a result of occupational injuries. The OHS questions that were included in the 2009 LFS questionnaire were based on questions from European and Scandinavian surveys on OHS, modified at a WAHSA workshop by OHS professionals from 8 SADC member states, and then further modified in consultation with CSO. Questionnaires were administered in the homes of the survey participants by trained research assistants in a language chosen by the interviewee.

### Data analysis

Data on 61871 employed persons out of a total of 129,765 respondents were available for analysis. Frequencies were used to estimate the magnitudes of occupational injuries. Logistic regression analysis was conducted in order to determine the degree of association between demographic, social and economic factors on one hand and injury and compensation on the other. Unadjusted odds ratios and adjusted odds ratios together with their 95% Confidence Intervals are reported.

## Results

### Socio-demographic description of the sample

Of the 61,871 study participants, 30,478 (49.2%) were males. The social, demographic and economic distributions of participants by gender are shown in Table 1. Overall, the age distribution between males and females was similar; males however tended to have never been married. While more male (56.1%) than female (37.6%) respondents were employers/self employed, a higher proportion of females (52.6%) were unemployed family workers compared to males (19.9%).

**Table 1:** Distribution of respondents by demographic, social and economic factors, Zambia 2009

Characteristic	Male n (%)	Female n (%)	Total n (%)
**Age (years)**			
5-9	345 (1.1)	285 (0.9)	630 (1.0)
10-14	847 (2.8)	719 (2.3)	1566 (2.5)
15-19	2300 (7.5)	2854 (9.1)	5154 (8.3)
20-24	3796 (12.5)	5327 (17.0)	9123 (14.7)
25-34	9542 (31.3)	9646 (30.7)	19188 (31.0)
35+	13648 (44.8)	12562 (40.0)	26210 (42.4)
Total	30478 (100)	31393 (100)	61871 (100)
**Marital status**			
Never married	7759 (26.0)	4977 (16.1)	12736 (21.0)
Married/cohabiting	20422 (68.4)	19495 (63.1)	39917 (65.7)
Separated/Divorced/Widowed	1665 (5.6)	6399 (20.7)	8064 (13.3)
Total	29846 (100)	30871 (100)	60717 (100)
**Province**			
Western	2947 (9.7)	2980 (9.5)	5927 (9.6)
Copperbelt	4629 (15.2)	3826 (12.2)	8455 (13.7)
Eastern	4231 (13.9)	4492 (14.3)	8723 (14.1)
Luapula	2871 (9.4)	3212 (10.2)	6083 (9.8)
Lusaka	3313 (10.9)	2949 (9.4)	6262 (10.1)
Northern	3939 (12.9)	4229 (13.5)	8168 (13.2)
North-Western	1867 (6.1)	2180 (6.9)	4047 (6.5)
Southern	3879 (12.7)	4200 (13.4)	8079 (13.1)
Central	2802 (9.2)	3325 (10.6)	6127 (9.9)
Total	30478 (100)	31393 (100)	61871 (100)
Current* employment status			
Unpaid family worker	5872 (19.9)	15382 (52.6)	21254 (36.2)
Paid employee	6962 (23.6)	2796 (9.6)	9758 (16.6)
Employer/self employed	16519 (56.1)	10976 (37.6)	27495 (46.9)
Other	87 (0.3)	74 (0.3)	161 (0.3)
Total	29440 (100)	29228 (100)	58668 (100)
**Number of employees in a workplace**			
5+	8831 (30.1)	5573 (19.2)	14404 (27.7)
< 5	20500 (69.9)	23519 (80.8)	44019 (75.3)
Total	29331 (100)	29092 (100)	58423 (100)
**Employer**			
Central government	1504 (5.1)	898 (3.1)	2402 (4.1)
Local government/Council	204 (0.7)	116 (0.4)	320 (0.5)
Parastatal/State owned firm	336 (1.1)	86 (0.3)	422 (0.7)
Private	6637 (22.5)	2971 (10.2)	9608 (16.4)
NGO or Church	219 n(0.7)	132 (0.5)	351 (0.6)
International organization	48 (0.2)	24 (0.1)	72 (0.1)
Household	20491 (69.6)	25003 (85.5)	45494 (77.5)
Total	29439 (100)	29230 (100)	58669 (100)

* status in the previous 7 days to the survey

NGO - Non Governmental Organization

Numbers not adding up due to missing information

### Injuries sustained at workplace

The distribution of injuries reported to have been sustained at work, by gender, is shown in Table 2. Overall, 8.1% of the respondents reported having sustained injuries arising from work in the past 12 months prior to the survey. The commonest type of injury was the "open wound" as reported by 81.6% of the respondents, with 80.5% and 83.3% among males and females, respectively. Of those injured, 60.5% reported having stayed away from work as a result, with no difference between males and females.

**Table 2:** Serious injuries suffered at workplace in the past 12 months prior to the survey by sex.

Factor	Male n (%)	Female n (%)	Total n (%)
**Suffered from any injuries due to** **work in past 12 months**			
Yes	3052 (10.0)	1946 (6.2)	4998 (8.1)
No	27426 (90.0)	29447 (93.8)	56873 (91.9)
Total	30478 (100)	31393 (100)	61871 (100)
**Most serious injury suffered due** **to work in past 12 months**			
Open wounds	2455 (80.5)	1621 (83.3)	4076 (81.6)
Fractures	239 (7.8)	109 (5.6)	348 (7.0)
Dislocations	108 (3.5)	51 (2.6)	159 (3.2)
Loss of limb/any part of the body	29 (1.0)	5 (0.3)	34 (0.7)
Loss of sight	15 (0.5)	13 (0.7)	28 (0.6)
Loss of hearing	11 (0.4)	9 (0.5)	20 (0.4)
Burns	116 (3.8)	99 (5.1)	215 (4.3)
Other	75 (2.5)	39 (2.0)	114 (2.3)
Total	3048 (100)	1946 (100)	4994 (100)
**Stayed away from work due to** **above injury**			
Yes	1891 (62.1)	1131 (58.1)	3022 (60.5)
No	1155 (37.9)	815 (41.9)	1970 (39.5)
Total	3046 (100)	1946 (100)	4992 (100)
**Days away from work due to** **above injury**			
< 7	637 (33.8)	419 (37.0)	1056 (35.0)
7-13	522 (27.7)	310 (27.4)	832 (27.6)
14-20	295 (15.7)	202 (17.9)	497 (16.5)
21+	430 (22.8)	200 (17.7)	630 (20.9)
Total	1884 (100)	1131 (100)	3015 (1000
**Received compensation from work** **as a result of the above injury**			
Yes	242 (8.0)	33 (1.7)	275 (5.5)
No	2796 (92.0)	1896 (98.3)	4692 (94.5)
Total	3038 (100)	1929 (100)	4967 (100)

Numbers not adding up due to missing information

In multivariate analysis (Table 3), all the factors considered in the analysis, except age and number of employees in a workplace were independently associated with serious injury. Being of male gender, married or married before, an employee of a private company or a household, and a paid employee were positively associated with serious injuries. Compared to persons who resided in the Western province, persons who resided in Copperbelt, Luapula, Northern and North-Western provinces were more likely to report serious injuries; while persons resident in the Eastern, Lusaka and Southern provinces were less likely to report serious injuries.

**Table 3:** Demographic, social and economic factors associated with serious injuries sustained at workplace

Factor	Total	Suffered serious injury n (%)	Bivariate OR* (95%cCI)	Multivariate AOR** (95%CI)
**Age (years)**				
5-9	630	12 (1.9)	1	-
10-14	1566	41 (2.6)	0.76 (0.58, 1.01)	
15-19	5155	153 (3.0)	0.87 (0.73, 1.03)	
20-24	9125	323 (3.5)	1.04 (0.90, 1.20)	
25-34	19188	991 (5.2)	1.54 (1.36, 1.75)	
35+	26213	1498 (5.7)	1.72 (1.52, 1.94)	
**Gender**				
Female	31393	1131 (3.6)	1	1
Male	30478	1887 (6.2)	1.33 (1.28, 1.38)	1.27 (1.21, 1.33)
**Current marital status**				
Never married	12736	416 (3.3)	1	1
Married/cohabiting	39919	2147 (5.4)	1.19 (1.13, 1.26)	1.11 (1.05, 1.17)
Separated/divorced/widowed	8064	430 (5.3)	1.18 (1.10, 1.27)	1.27 (1.17, 1.38)
**Province**				
Western	6128	241 (3.9)	1	1
Copperbelt	8455	489 (5.8)	1.23 (1.12, 1.34)	1.13 (1.02, 1.24)
Eastern	8723	197 (2.3)	0.46 (0.41, 0.53)	0.49 (0.43, 0.56)
Luapula	6083	507 (8.3)	1.82 (1.67, 1.99)	1.90 (1.73, 2.08)
Lusaka	6264	230 (3.7)	0.76 (0.68, 0.86)	0.70 (0.62, 0.81)
Northern	8168	449 (5.5)	1.17 (1.06, 1.28)	1.20 (1.09, 1.32)
North-Western	4049	337 (8.3)	1.82 (1.64, 2.02)	1.81 (1.62, 2.02)
Southern	8080	307 (3.8)	0.79 (0.71, 0.88)	0.77 (0.69, 0.86)
Central	5929	261 (4.4)	0.82 (0.73, 0.92)	0.90 (0.80, 1.02)
**Current employment status**				
Unpaid family worker	21272	693 (3.3)	1	1
Paid employee	9770	581 (5.9)	1.22 (1.15, 1.30)	1.27 (1.15, 1.40)
Employer/self employed	27521	1686 (6.1)	1.26 (1.20, 1.33)	1.02 (0.96, 1.09)
**Number of employees in a workplace**				
5+	14416	774 (5.4)	1	-
< 5	44064	2189 (5.0)	0.96 (0.92, 1.00)	
**Employer**				
Central government	2402	62 (2.6)	1	1
Local government/Council	321	19 (5.9)	1.38 (0.87, 2.18)	1.27 (0.80, 2.01)
Parastatal/State owned firm	424	27 (6.4)	1.49 (0.99, 2.23)	1.33 (0.89, 2.01)
Private	642	642 (6.7)	1.57 (1.22, 2.00)	1.61 (1.25, 2.07)
NGO or Church	351	12 (3.4)	0.78 (0.45, 1.33)	0.82 (0.47, 1.44)
International organization	72	2 (2.8)	0.63 (0.19, 2.10)	0.60 (0.18, 2.03)
Household	2199	2199 (4.8)	1.11 (0.87, 1.41)	1.42 (1.09, 1.85)

* OR Odds ratios

** AOR Adjusted Odds ratio

NGO - Non Governmental Organization

### Compensation patterns

Overall, only 5.5% of the participants were compensated, with 8.0% among males and 1.7% among females (Table 2). Table 4 shows demographic, social and economic factors associated with receiving compensation for work-related injuries. Compensation varied by gender, geographical area (province), employment status, and number of employees stationed in a workplace.

**Table 4:** Demographic, social and economic factors associated with receiving compensation for work-related injury or other disease

Factor	Total	Received compensation n (%)	Bivariate OR (95%cCI)	Multivariate AOR (95%CI)
**Age (years)**				
< 15	166	4 (2.4)	1	-
15-19	489	15 (3.1)	0.79 (0.50, 1.25)	
20-24	1127	44 (3.9)	1.01 (0.73, 1.41)	
25-34	3109	195 (6.3)	1.66 (1.28, 2.16)	
35+	4709	223 (4.7)	1.24 (0.96, 1.60)	
**Gender**				
Female	4122	85 (2.1)	1	1
Male	5478	396 (7.2)	1.92 (1.71, 2.17)	1.52 (1.22, 1.90)
**Current marital status**				
Never married	1343	84 (6.3)	1	-
Married/cohabiting	6770	349 (5.2)	1.10 (0.96, 1.27)	
Separated/divorced/widowed	1414	45 (3.2)	0.67 (0.54, 0.83)	
**Province**				
Western	605	23 (3.8)	1	1
Copperbelt	1397	197 (14.1)	4.04 (3.39, 4.83)	2.32 (1.77, 3.05)
Eastern	612	30 (4.9)	1.27 (0.90, 1.79)	1.25 (0.75, 2.09)
Luapula	1621	35 (2.2)	0.54 (0.40, 0.75)	0.63 (0.35, 1.14)
Lusaka	798	85 (10.7)	2.94 (2.33, 3.70)	1.31 (0.89, 1.93)
Northern	1416	19 (1.3)	0.34 (0.22, 0.51)	0.67 (0.40, 1.12)
North-Western	1265	39 (3.1)	0.78 (0.58, 1.06)	1.64 (1.03, 2.61)
Southern	1018	33 (3.2)	0.83 (0.59, 1.15)	0.70 (0.44, 1.11)
Central	868	20 (2.3)	0.58 (0.39, 0.87)	0.79 (0.41, 1.52)
**Current employment status**				
Unpaid family worker	2401	39 (1.6)	1	1
Paid employee	1716	299 (17.4)	4.84 (4.17, 5.60)	3.46 (2.59, 4.62)
Employer/self employed	5236	122 (2.3)	0.55 (0.46, 0.65)	0.64 (0.48, 0.84)
**Number of employees in a workplace**				
5+	2379	272 (11.4)	1	1
< 5	6970	189 (2.7)	0.47 (0.42, 0.51)	0.75 (0.63, 0.89)
**Employer**				
Central government	277	38 (13.7)	1	-
Local government/Council	56	8 (14.5)	1.07 (0.54, 2.11)	
Parastatal/State owned firm	74	16 (21.6)	1.73 (1.01, 2.95)	
Private	1879	239 (12.7)	0.91 (0.69, 1.21)	
NGO or Church	37	5 (13.5)	0.98 (0.42, 2.26)	
International organization	12	5 (-)	4.47 (1.64, 12.20)	
Household	7043	150 (2.1)	0.14 (0.10, 0.18)	

* Confidence interval does not include 1

NGO - Non Governmental Organization

(-) Percent not reported because denominator is less than 30

## Discussion

Data on occupational injuries is scarce and unreliable in Zambia, and it is therefore difficult for stakeholders (Government, employees or employers) to estimate their health and socio-economic impacts and target or assess the efficacy of interventions. While data is routinely collected in Zambia through accident notifications to the Ministry of Labour and Social Security, and Worker's Compensation Fund, there is widespread underreporting of cases, and until now there has not been regular system of supplementary or corroborative data such as the annual surveys carried out in Scandinavia or elsewhere [9]. Only 14 fatal occupational injuries were reported to the International Labour Organisation (ILO) in 2000, and none in 2005. ILO estimates for 2005 were, for an economically active population of 4.4 million, 788 (0.18 per 1000) fatal accidents, and 601,399 (13.7%) >3 day accidents [10].

The labour force survey is one of the largest regular household surveys conducted in Zambia. The results from the 2009 LFS indicate that 8.1% of workers reported having been injured in the past 12 months, and 60.5% reported having been absent from work because of workplace injuries. The injury rate is less than what was reported in the 2005 LFS of 20.6% among persons of age 15 years or older [8], partly because of differences in the age groups studied; and partly because a weighted analysis was used in the 2005 LFS and not in the 2009 LFS. The injury rate is also somewhat less than the ILO estimate of 13.7% for 3 day injuries for 2005 [10]: this may be linked to the other finding, discussed below, that few workers, reported having been compensated for their injuries; if they were not to be paid for time taken off work, injured workers may have continued working despite their injuries when they would otherwise have remained at home. These figures do indicate that occupational injury has a significant health and economic impact on working life of Zambians, and provide a baseline against which to measure future trends in occupational injury, and measure the impact of future interventions on occupational injury rates.

There are gender differences in the distribution of reported injury. Men were 27% more likely to report having suffered serious injuries than women, and this difference has been reported elsewhere. In the National Health Interview Survey (NHIS) in the United States, Forrest and Cali [11] reported that men had more than four-times higher rate of eye injury at work than women. However, our results indicate that women workers in Zambia are disadvantaged in several respects, compared to their male counterparts. Female respondents tended to be married or were once married, and to work as unpaid family workers. Although 60.5% of all injured participants reported staying away from work because of workplace injury, only 5.5% of the respondents received compensation.

We found that workers who were paid employees were more likely to be injured than unpaid family workers. There are several possible reasons why this may be the case. Firstly, it is possible that unpaid family workers may have some control over their environments if working in their own homes. Secondly, it is possible the unpaid family workers may be more liberal in excusing themselves from work due to injury while it may be harder for those who are paid employees. Finally, it is also possible that the nature of the jobs in unpaid family workers may have been different from those in paid employment such that the nature and severity of injuries may have been different.

Finally, our findings indicate that workers in specific geographic areas of Zambia, and employed by private companies or households, are particularly at risk of occupational injury. This information is valuable for the introduction of targeted interventions to reduce injury rates where they are highest, and is information that the Zambian Ministry of Labour has not previously had at its disposal.

## Limitations of the study

The present study has a number of limitations. Firstly, data were collected via self-reports. To the extent that study participants misreported either intentionally or unintentionally, our findings may have suffered from misclassification. If for instance there was a difference in the extent of misreporting between males and females, or between old or younger workers, our results may have been biased. We however do not have information to suggest that such may have been the case. Secondly, the questionnaire was not translated into local languages. Although interviewers were trained in how to administer the questionnaire in local languages, the questions may not have been standardized. The correct procedure is to have one expert translate it into a local language and another expert independently translates it back to English. However, we believe that the bias that might have been introduced into our findings was minimal because CSO uses experienced interviewers. Thirdly, since the design of the data collection was cross sectional, it is not possible to assign causation to any of the explanatory variables. We also did not explore what other factors, e.g., underlying medical conditions [12], stress [13], and physical disabilities may be associated with experiencing an occupational injury. Furthermore, we produced unweighted estimates because we were unable to compute weights due to lack of information on response rates and selection probabilities; and our estimates may be biased to the extent of non-respondents.

## Conclusion

Data generated through the addition of OHS related questions to a routine household survey in Zambia provide useful information on the distribution and determinants of occupational injury, as has been reported from the addition of OHS related questions to national household surveys in South Africa [14]. The prevalence of reported injury, and its association with a significant level of absence from work, indicates that occupational hazards in Zambia have significant health and economic effects not indicated by the official statistics. The data provide good social and socio-economic grounds to initiate an improvement in working conditions to prevent these occurrences as well as a baseline on which to base statistical targets for improvement. There was geographic variation in the distribution of reported injury, with higher reported prevalence in specific provinces. This information could be useful to the Ministry of Labour to identify areas in specific need of attention, especially in terms of surveillance.

## Competing interests

The authors declare that they have no competing interests.

## Authors' contributions

SS analysed the data, interpreted the results and participated in the drafting of the manuscript. ASM participated in the interpretation of the results and led the drafting of the manuscript. AR participated in the designing of the questionnaire, interpretation of results and drafting of the manuscript. ER participated in the interpretation of the results and drafting of the manuscript. All authors read and approved the final manuscript.
